# A missense variant in Exon 9 of the *ASNS* gene causes splicing abnormality in an Infant with asparagine synthetase deficiency

**DOI:** 10.3389/fgene.2026.1799796

**Published:** 2026-05-28

**Authors:** Weihong Zhang, Fengliu Chen, Zuo Wang, Han Ding, Yu Zhang, Lingkong Zeng

**Affiliations:** 1 Department of Neonatology, Wuhan Children’s Hospital, Tongji Medical College, Huazhong University of Science & Technology, Wuhan, China; 2 Medical College, Faculty of Medicine, Wuhan University of Science and Technology, Wuhan, China

**Keywords:** ASNS, asparagine synthetase deficiency, epilepsy, hypotonia, microcephaly, whole-exome sequencing

## Abstract

Asparagine Synthetase Deficiency (ASNSD) is a rare neurodevelopmental disorder primarily caused by pathogenic homozygous or compound heterozygous variants in the *ASNS* gene. Clinical manifestations typically include microcephaly, severe psychomotor developmental delay, progressive encephalopathy, epilepsy, and other associated neurological abnormalities. Herein, we performed a comprehensive clinical assessment of a neonate presenting with microcephaly and hypotonia from a Chinese family. Whole-exome sequencing (WES) identified two novel compound heterozygous variants in the *ASNS* gene: c.1031-6_1041del (paternally inherited) and c.1049A>G (maternally inherited). The c.1031-6_1041del variant disrupts the canonical splice region and was classified as likely pathogenic. Notably, the c.1049A>G variant, localized to Exon 9, results in a missense substitution (p.Lys350Arg) and was predicted by *in silico* tools to induce aberrant splicing. Furthermore, functional validation via *in vitro* splicing assays confirmed that this missense variant leads to partial deletion of Exon 9, thereby possibly impairing the normal expression and function of the *ASNS* gene. These findings expand the mutational spectrum of ASNSD and provide critical insights into the pathogenic mechanisms underlying splicing dysregulation caused by missense variants in the *ASNS* gene.

## Introduction

Asparagine Synthetase Deficiency is a rare autosomal recessive neurodevelopmental disorder with a global incidence of <1/1,000,000 (Liu et al., 2022). It is caused by defects in the *ASNS* gene, which encodes asparagine synthetase (Gupta et al., 2017; Alfadhel et al., 1993). Clinically, ASNSD is characterized by congenital microcephaly, severe developmental delay, central hypotonia, and refractory epilepsy (Gataullina et al., 2016; Ruzzo et al., 2013; Lomelino et al., 2017). Notably, this disease carries a poor prognosis: a significant time lag often exists between symptom onset and definitive diagnosis (Song et al., 2024), and approximately half of affected patients succumb within the first year of life (Ben-Salem et al., 2015; Chang et al., 2023).

Functionally, asparagine synthetase plays a pivotal role in asparagine biosynthesis, catalyzing the conversion of aspartic acid to asparagine using glutamine as the amino donor in an ATP-dependent reaction (Alfadhel et al., 1993; Sun et al., 2017; Alfadhel et al., 2015). Pathogenic *ASNS* variants disrupt the structure or function of asparagine synthetase, leading to intracellular asparagine depletion and subsequent inhibition of cell growth (Staklinski et al., 2023).

Notably, *ASNS* has been demonstrated to be critical for brain development in murine models (Ruzzo et al., 2013). Cells primarily acquire Asn via *de novo* biosynthesis, and *ASNS* is the sole enzyme responsible for catalyzing this biosynthetic process (Chang et al., 2023).

The *ASNS* gene is localized to chromosome 7q21.3, and consists of 13 exons (Zhang et al., 1989; Heng et al., 1994), and relies on the cooperative action of its two functional domains to complete the entire biosynthetic reaction (Sacharow et al., 2018; Richards and Kilberg, 2006).

Since its first description in 2013, approximately 60 families and over 100 patients with ASNSD have been reported worldwide to date, alongside 60 distinct pathogenic variants in the *ASNS* gene (Palmer et al., 2015). Of these, missense variants account for 73% of all known pathogenic variants (Jahanpanah et al., 2024), highlighting their predominant role in disease pathogenesis.

In the present study, we identified two novel *ASNS* variants—c.1031-6_1041del and c.1049A>G—in a neonate diagnosed with ASNSD from a Chinese family. Neither variant has been previously documented in public human genetic databases (e.g., gnomAD, ClinVar). Functional validation via *in vitro* splicing assays confirmed the pathogenicity of the missense variant (c.1049A>G). Collectively, this study expands the clinical and mutational spectrum of ASNSD, providing additional insights for the genetic diagnosis and counseling of this rare disorder.

## Methods

### Participants

One neonate presenting with microcephaly, hypotonia, and seizures, diagnosed with Asparagine Synthetase Deficiency, was enrolled in this study. Peripheral blood samples were collected from the patient and their parents. This study was conducted in accordance with the ethical standards of the Declaration of Helsinki and approved by the Ethics Committee of Wuhan Children’s Hospital (Wuhan Maternal and Child Healthcare Hospital), Tongji Medical College, Huazhong University of Science and Technology (Approval No.: 2025R06-E01). Written informed consent was obtained from the family members of all participating patients.

### Whole-exome sequencing (WES)

WES was performed to capture and sequence all coding regions of approximately 20,000 human genes and their proximal flanking regions (∼20 bp) using genomic DNA isolated from samples, targeted enrichment, and high-throughput sequencing. The detailed procedures were as follows: Genomic DNA was extracted from peripheral blood samples using the QIAamp Blood DNA Mini Kit (Cat No./ID: 51104), and its concentration and purity were measured using a NanoDrop 2000 spectrophotometer. DNA was fragmented to a length range of 150–300 bp using a Covaris M220 ultrasonicator. The fragmented DNA underwent end repair and A-tailing, followed by adapter ligation and amplification. Targeted capture of the amplified libraries was performed using the xGen® Exome Research Panel v1.0 (Integrated DNA Technologies, IDT). Paired-end 150 bp (PE150) sequencing was conducted on an Illumina NovaSeq 6000 platform. Data analysis was performed using an in-house pipeline. After sequence alignment, we applied an allele frequency threshold of <1% in the East Asian subpopulation as the primary filtering criterion, retaining only variants with a frequency below 1% in the East Asian population from the gnomAD v2.1.1 database. The variants were then prioritized based on the relevance of the associated genes to the clinical phenotypes, followed by pathogenicity assessment according to the American College of Medical Genetics and Genomics (ACMG) standards and guidelines for the interpretation of sequence variants.

### Sanger sequencing

Sequences of the target regions were downloaded from the UCSC Genome Browser, with the reference genome version consistent with the physical location of the gene of interest. Specific primers for the regions to be amplified were designed using Primer Premier 5 or Primer3Plus software and synthesized by Sangon Biotech (Shanghai) Co., Ltd. PCR amplification was performed using the TaKaRa LA PCR™ Kit Ver.2.1. After amplification, 2 μL of PCR products were subjected to 1% agarose gel electrophoresis for quality control at 140 V for 20 min. Qualified PCR products were sent to Sangon Biotech (Shanghai) Co., Ltd. for Sanger sequencing. Sequencing data were analyzed using BioEdit software after retrieval.

### Minigene assay

To investigate whether the identified variants affect mRNA splicing, a minigene assay was performed. The detailed procedures were as follows: First, two recombinant minigene vectors (pcDNA3.1 and pcMINI) were constructed (Supplementary Figure S1). Second, the recombinant vectors were transiently transfected into 293T and HeLa cell lines. Subsequently, total RNA was extracted from the transfected cells and reverse-transcribed into cDNA. Finally, PCR amplification was performed using primers located on the vectors, and the amplified gene transcription bands were detected by agarose gel electrophoresis. Detailed protocols are provided in Supplementary Data S1.

## Results

### Clinical manifestations and imaging findings of the proband

The proband was a 6-month-old male infant. His parents denied consanguineous marriage. The father and the first child were healthy, while the mother had intellectual disability, and no family history of genetic diseases was reported. The mother conceived naturally without regular prenatal examinations. As the third fetus and second live birth, the proband was a preterm infant born at 37 weeks and 5 days of gestation via cesarean section due to abnormal fetal heart rate and fetal movement. His birth weight was 2.3 kg, with Apgar scores of 8 at 1 min and 8 at 5 min. Immediately after birth, he was admitted to the Neonatal Intensive Care Unit (NICU) of a local hospital due to poor responsiveness and limb twitching. At 7 days of age, the proband was transferred to the NICU of Wuhan Children’s Hospital due to recurrent limb twitching.

**FIGURE 1:**
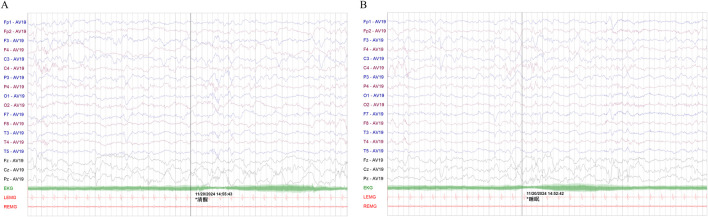
Pre-treatment EEG. From left to right: wakefulness period, sleep period. Abnormal electroencephalogram, characterized by multifocal and a large number of sharp waves, spike waves, and spike-slow waves.

**FIGURE 2:**
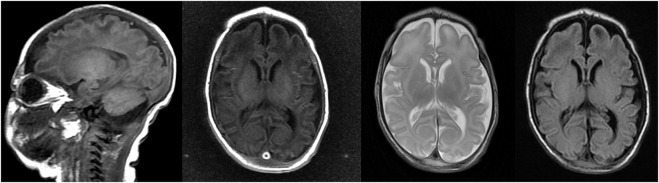
Cranial MRI. From left to right: T1 FLAIR, T2 plain scan, T2 FLAIR. Bilateral frontoparietal white matter shows patchy slightly long T1 and long T2 signals on T1WI and T2WI sequences, and hypointense signals on FLAIR sequence. The left lateral ventricle is slightly enlarged, with widened bilateral sylvian fissures, anterior longitudinal fissure, and frontal extracerebral spaces. The corpus callosum is relatively thin.

At birth, he presented with microcephaly (head circumference: 28.00 cm), a birth weight of 2.3 kg, and a body length of 44.0 cm, accompanied by hypotonia, poor responsiveness, and weak crying. However, primitive reflexes such as sucking and rooting could be elicited. Mild feeding difficulties were noted, with slow sucking. At 1 month of age, he developed focal seizures characterized by left facial twitching, bilateral lower limb twitching, salivation, and loss of consciousness, with each episode lasting 1 to more than 10 min and 3-4 episodes per day. Magnetic Resonance Imaging (MRI) and electroencephalogram (EEG) examinations revealed abnormal brain structure and epileptiform discharges (Figures 1, 2). Following combined treatment with levetiracetam (0.3 mL/dose, twice daily) and oxcarbazepine (2 mL/dose, twice daily), seizure frequency decreased, with a seizure-free period of 1 month. However, seizures recurred at 5 months of age with the same semiology, indicating drug-resistant epilepsy. During treatment, the patient was diagnosed with congenital laryngomalacia and experienced recurrent hospitalizations due to pneumonia. At 6 months of age, the proband was still unable to lift his head, fix his gaze, or sit independently. He was lost to follow-up after 6 months of age. Routine laboratory tests, including urine and blood examinations, showed no abnormalities in urinary organic acids, blood amino acids, or acylcarnitines (Supplementary Table S3).

### Whole-exome sequencing and sanger sequencing

**FIGURE 3:**
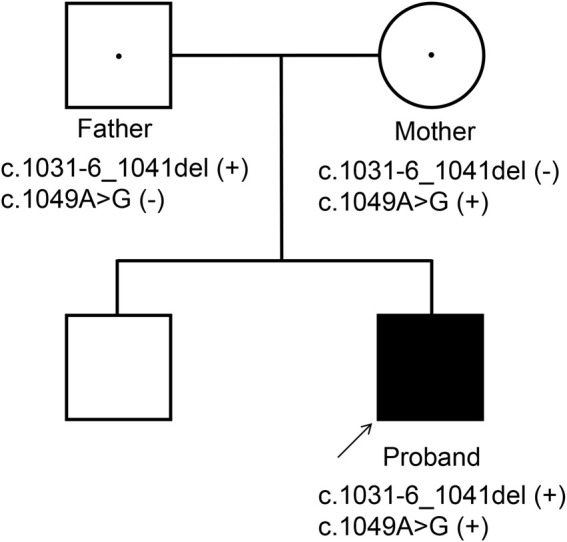
Pedigree chart of the family carrying *ASNS* gene variants.

**FIGURE 4:**
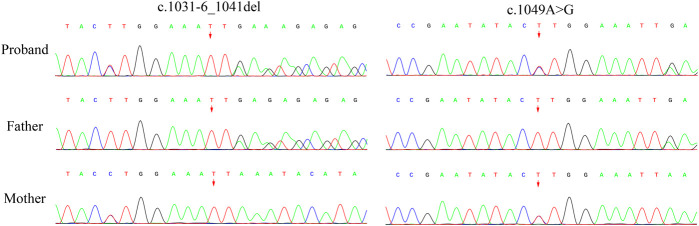
Sanger sequencing results. Variants c.1031-6_1041del and c.1049A>G were inherited from the father and mother, respectively.

To explore the potential genetic etiology of the disease, WES was performed on the proband. After completion of whole-exome sequencing, the sequencing quality of the sample was assessed. The results showed that the average sequencing depth was 145.65×, and the percentage of target exonic regions achieving a depth of ≥20× was 98.24%. The library alignment rate was 99.99%, the Q30 score was 96.84%, and the capture efficiency was 84.90%, indicating that the sequencing data were of high quality and met the requirements for subsequent variant analysis. WES identified two compound heterozygous variants in the *ASNS* gene (NM_001673.5) of the proband: c.1031-6_1041del and c.1049A>G, which were inherited from his father and mother, respectively (Figure 3). Further Sanger sequencing validation confirmed that c.1031-6_1041del was inherited from the father, while c.1049A>G was inherited from the mother (Figure 4).

We also analyzed the copy number of the *ASNS* gene in the whole-exome sequencing data. No copy number alterations in this gene were identified in the proband compared with other tested samples (Supplementary Figure S2).

#### According to the ACMG guidelines

The c.1031-6_1041del variant was classified as likely pathogenic (PVS1 + PM2_Supporting). Rationale: Loss-of-function (LOF) is the established pathogenic mechanism for the disease, and this variant is predicted to be a null variant (PVS1); the variant is absent in normal control populations from the ESP, 1000 Genomes Project, and ExAC databases (PM2_Supporting).

The c.1049A>G variant was classified as variant of uncertain significance (PM2_Supporting + PM3). Rationale: The variant is absent in normal control populations from the ESP, 1000 Genomes Project, and ExAC databases (PM2_Supporting); a pathogenic variant was detected in trans in this autosomal recessive disorder (PM3).

### *In vitro* splicing assay

Multiple *in silico* prediction tools indicated that both c.1031-6_1041del (located in the canonical splice region) and c.1049A>G (located in an exon) would cause aberrant mRNA splicing (Table 1). To further investigate the pathogenicity of c.1049A>G, two minigene vectors were constructed to assess its potential functional impact. Wild-type and mutant expression vectors (pcDNA3.1 and pcMINI) containing Exon 9 were transfected into 293T and HeLa cell lines for splicing analysis, respectively.

**TABLE 1:** Genotypes and protein function prediction of *ASNS* variants.

Position	Variant	Protein	Inherited		Prediction	
				spliceAI	RDDC	FF
chr7:97484761	c.1031-6_1041del	p.?	Father	1.00 (Acceptor loss)	0.9552 (Acceptor gain)	0.94 (Acceptor loss)
chr7:97484753	c.1049A>G	p.Lys350Arg	Mother	0.99 (Acceptor gain)	0.9975 (Acceptor gain)	0.93 (Acceptor gain)

**FIGURE 5:**
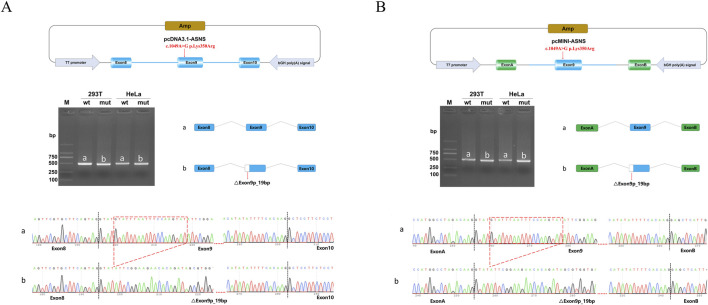
Minigene assay results. **(A)** Results of pcDNA3.1 vector experiment: both 293T and Hela cell lines showed a 19bp deletion in Exon 9. **(B)** Results of pcMINI vector experiment: similarly, a 19bp deletion in Exon 9 was observed in both 293T and Hela cell lines.

Normal splicing patterns were observed in cells transfected with wild-type vectors, regardless of the vector type (pcDNA3.1 or pcMINI) or cell line (293T or HeLa). In contrast, cells transfected with mutant vectors showed a 19 bp deletion in the left region of Exon 9 (Figure 5), which was validated by Sanger sequencing. This deletion resulted in partial loss of Exon 9, denoted as c.1031_1049del (p.Met345IlefsTer10) at the cDNA and protein levels. The 19 bp deletion in Exon 9 caused a frameshift, leading to the generation of a premature termination codon (PTC) within Exon 9 and potentially producing a truncated protein of 353 amino acids. Unfortunately, we were unable to obtain cellular samples from the patient to validate this finding. Consequently, the variant can only be classified as a variant of uncertain significance.

### Overview of reported *ASNS* variants

A literature search was performed from 2013 to 2025. Chinese databases (CNKI and Wanfang Data) were searched using the keywords “asparagine synthetase gene”, “Asparagine Synthetase Deficiency”, and “*ASNS*”. The PubMed database was searched using the keywords “*ASNS*” and “Asparagine Synthetase Deficiency”. A total of 41 relevant studies were retrieved. To date, 100 ASNSD patients from 63 distinct families with pathogenic *ASNS* variants have been confirmed, including the proband in this study (Supplementary Table S2).

Clinical manifestations of these patients are concentrated in microcephaly, intellectual disability, and developmental delay, epilepsy, abnormal muscle tone, as well as abnormal EEG and cranial MRI findings. Among them, there were 65 male patients, 33 female patients, and 2 patients with unknown gender. Microcephaly was reported in 92% of patients (92/100), intellectual disability and developmental delay in 99% (99/100), and epilepsy in 69% (69/100). In addition, abnormal muscle tone, abnormal EEG, and abnormal cranial MRI are also common clinical features.

To date, 71 distinct pathogenic *ASNS* variants have been identified, including 48 missense variants, 2 nonsense variants, 6 frameshift variants, and 4 splice region variants. Notably, no previously reported compound heterozygous variants consisting of a missense variant and a splice region variant were found, which is distinct from the variant combination (c.1049A>G, missense variant; c.1031-6_1041del, splice region variant) identified in our study.

## Discussion

In this study, we conducted genetic evaluation of an infant diagnosed with Asparagine Synthetase Deficiency. The patient presented with microcephaly, mild hypotonia, diminished primitive reflexes, dyspnea, feeding difficulties, epilepsy, developmental delay, congenital laryngomalacia, abnormal brain structure and epileptiform discharges on electroencephalogram (EEG), which were highly consistent with the clinical manifestations reported in almost all ASNSD patients to date. Whole-exome sequencing (WES) identified two novel variants in the *ASNS* gene: c.1049A>G (p.Lys350Arg) and c.1031-6_1041del. The proband inherited these variants in a compound heterozygous state from his parents, consistent with the autosomal recessive inheritance pattern of ASNSD. This study expands the mutation spectrum of the *ASNS* gene.

A key finding of this study is that the c.1049A>G variant disrupts normal splicing patterns. Splicing is a critical regulatory step in gene expression, involving the excision of introns and ligation of exons. Exon-intron boundaries are defined by conserved sequences at the 3′and 5′splice sites, which facilitate exon recognition (Marasco and Kornblihtt, 2023). Alternative splicing is ubiquitous in eukaryotes, affecting a large proportion of mammalian genes and multiple regulatory mechanisms, and is of great significance in the context of inherited diseases and tumorigenesis. Notably, most splicing-related variants are localized to canonical splice regions, while exonic variants causing aberrant splicing are relatively rare. Our results demonstrated that the missense variant c.1049A>G (p.Lys350Arg) impairs the normal splicing of *ASNS* mRNA, with consistent findings across two independent vector systems (pcDNA3.1 and pcMINI). Specifically, this variant leads to a single aberrant splicing event: a 19 bp deletion in the left region of Exon 9. This variant is predicted to induce nonsense-mediated mRNA decay (NMD).

Splice variants in the *ASNS* gene are rare. The c.1476 + 1G>A variant was identified in two deceased siblings, and the aberrant splicing pattern caused by this variant is complex. Notably, no normal splicing products were detected in the analyzed samples. Both siblings presented with microcephaly and cerebellar hypoplasia, but without epileptic seizures (Akesson et al., 2020). Among other reported cases, patients harboring the c.1137 + 1G>A variant exhibited cortical blindness in addition to microcephaly, developmental delay, and epileptic seizures. In contrast, individuals carrying the c.674-1G>A variant showed a phenotype similar to our patient, with no additional abnormalities (Alharby et al., 2020). In contrast to the typical clinical features, our patient also presented with congenital laryngomalacia and recurrent pneumonia. Based on our statistical analysis of previously reported patients, these findings appear to be uncommon manifestations in ASNSD (Supplementary Table S2). We speculate that laryngomalacia-like features may arise secondarily from chronic hypotonia. Similarly, pneumonia is more likely a secondary infectious complication rather than a direct consequence of deficient *ASNS* function.

However, this study has certain limitations. We have identified only one patient with ASNSD so far, which limits the generalizability of our findings. Although we validated the pathogenic mechanism of the c.1049A>G variant via *in vitro* minigene assays, due to interruption of follow-up, we were unable to get patient-derived specimens to directly verify whether the truncated transcript undergoes nonsense-mediated mRNA decay (NMD) or whether the truncated protein is expressed in the proband. Additionally, functional studies such as asparagine concentration detection and enzyme activity assays were not performed, which could further confirm the loss-of-function effect of the identified variants. Therefore, we can only speculate that this variant may lead to abnormalities in the function of the *ASNS* gene, which requires further investigation in subsequent studies.

In conclusion, the novel compound heterozygous variants c.1031-6_1041del and c.1049A>G in the *ASNS* gene are likely the genetic basis of the proband’s clinical phenotype. The confirmation of aberrant splicing via *in vitro* functional analysis enhances the diagnostic accuracy for this patient. Future studies should focus on expanding the cohort of ASNSD patients, combining animal models and comprehensive molecular functional assays to elucidate the role of *ASNS* variants in the pathogenesis of ASNSD, as well as the physiological function of the *ASNS* gene in nervous system development. Given that adult carriers of *ASNS* pathogenic variants may be underrecognized, enhanced genetic screening in adult patients with developmental disorders is recommended to improve epidemiological data. Genetic testing plays a crucial role in the early identification of ASNSD, the formulation of appropriate treatment plans, and the provision of informed genetic counseling for affected families.

## Data Availability

Due to participant/patient confidentiality, the dataset of this article is not publicly available. Requests to access the dataset should be directed to the corresponding author.

## Supplementary material

SUPPLEMENTARY FIGURE 1 Construction maps of pcDNA3.1 and pcMINI vectors.

SUPPLEMENTARY FIGURE 2 Copy number analysis of the *ASNS* gene.

SUPPLEMENTARY TABLE 1 All primers used in vector construction.

SUPPLEMENTARY TABLE 2 Literature reported cases of all ASNSD patients.

SUPPLEMENTARY TABLE 3 Results of routine laboratory tests.
