# Supplementary figures and images for: A missense variant in Exon 9 of the ASNS gene causes splicing abnormality in an Infant with asparagine synthetase deficiency
Source: Front Genet. 2026 May 28;17:1799796. doi: 10.3389/fgene.2026.1799796 (PMC13252912; doi:10.3389/fgene.2026.1799796)

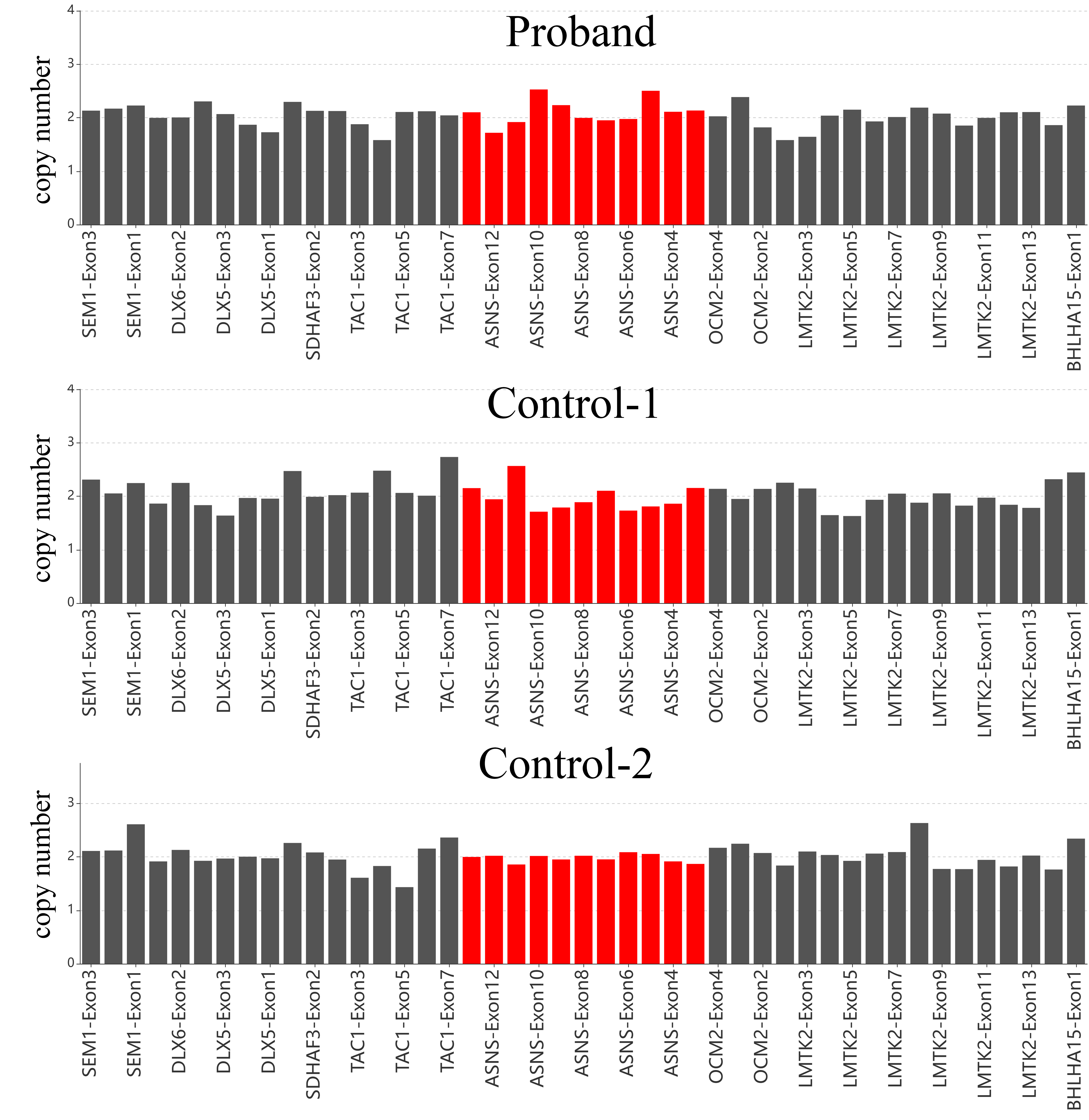

Supplement: Supplementary file 3 [file Image2.tif]

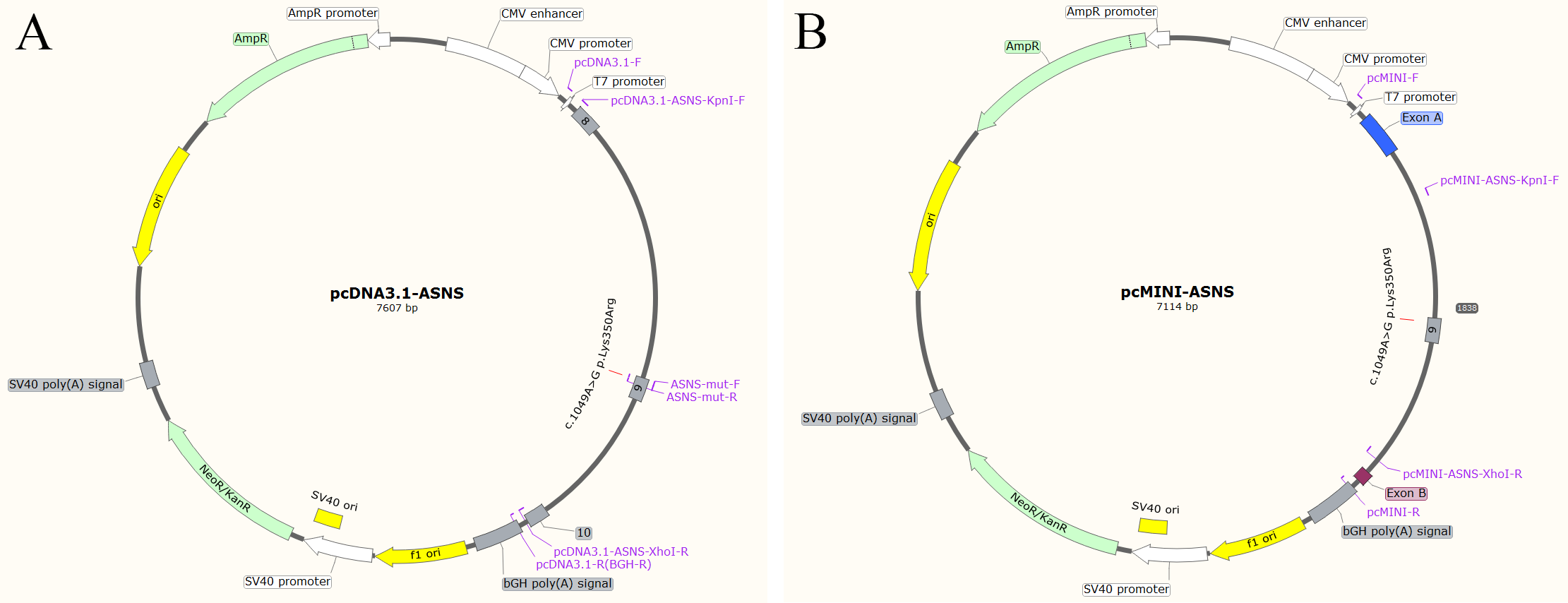

Supplement: Supplementary file 4 [file Image1.tif]
