# Supplementary material for: A missense variant in Exon 9 of the ASNS gene causes splicing abnormality in an Infant with asparagine synthetase deficiency
Source: Front Genet. 2026 May 28;17:1799796. doi: 10.3389/fgene.2026.1799796 (PMC13252912; doi:10.3389/fgene.2026.1799796)
Supplement: Supplementary file 6 [file DataSheet1.docx]

**Construction of Recombinant Vectors**

The construction of recombinant vectors included restriction enzyme digestion, ligation, transformation, and verification of recombinant clones. First, the target DNA fragment was obtained by nested PCR. The digestion reaction at 37°C (45 minutes, 30 μL) contained the following components: 2 μL of 10×NEB buffer, 0.6 μL of Enzyme 1, 0.6 μL of Enzyme 2, 500 ng/25 μL of vector/DNA fragment, and double-distilled water (ddH₂O) to make up the total volume to 30 μL. The ligation reaction (10 μL) consisted of: 1 μL of 10×ligase buffer, 7 μL of digested DNA fragment (wt/mut), 1 μL of digested vector, and 1 μL of ligase. Ligation was performed at 22°C for 90 minutes, and the ligation product was transformed into DH5α competent cells. The cells were cultured overnight at 37°C, and several single colonies were randomly selected for verification. Verification methods included colony/bacterial solution PCR and Sanger sequencing. The vector map is shown in Supplementary Figure 1.

**Cell Transfection**

Wild-type and mutant minigenes were inserted into pcDNA3.1 and pcMINI vectors respectively, resulting in 4 recombinant vectors. These vectors were transfected into HeLa and 293T cell lines separately. Transfection steps were performed according to the liposome instruction manual (Rapid Plasmid Mini Kit, 1005250, SIMGEN), and cell samples were harvested after 48 hours.

**Transcription Analysis**

Total RNA was extracted from cell samples, and equal amounts of RNA were used for reverse transcription to synthesize cDNA. Subsequently, pcDNA3.1-wt/mut was amplified by PCR using primers pcDNA3.1-F/pcDNA3.1-R, and pcMINI-wt/mut was amplified using primers pcMINI-F/pcMINI-R. Agarose gel electrophoresis was used to detect the gene transcription bands generated by amplification, and each band was recovered for Sanger sequencing. All primer sequences are listed in Supplementary Table 1.
